# Umbilicus Porocarcinoma: An Uncommon Neoplasm at a Unique Site

**DOI:** 10.7759/cureus.95343

**Published:** 2025-10-24

**Authors:** Inês Sousa, João Nobre, Paulo Clara, Nuno G Rama

**Affiliations:** 1 General Surgery, Local Health Unit of the Region of Leiria (Unidade Local de Saúde da Região de Leiria), Leiria, PRT; 2 General and Colorectal Surgery, Local Health Unit of the Region of Leiria (Unidade Local de Saúde da Região de Leiria), Leiria, PRT; 3 General Surgery, Local Health Unit of Coimbra (Unidade Local de Saúde de Coimbra), Coimbra, PRT

**Keywords:** dermathology, eccrine porocarcinoma, excision surgery, tumor, umbilicus

## Abstract

Eccrine porocarcinoma is a rare and aggressive cutaneous neoplasm that mainly affects the elderly patients. It occurs more often in the head and neck, followed by lower limbs. The abdomen, especially the umbilicus, is a very rare presenting location. Diagnosis can be challenging and is usually made based on a combination of clinical, dermoscopical and histopathological findings. The main treatment approach is wide surgical resection, with negative margins. Despite resection, development of nodal or distant metastasis occurs in a few cases. Adjuvant treatment modalities consist of radiotherapy and chemotherapy, but the results are inconsistent. Immunotherapy is a novel option to treat these cases, but more studies are needed.

Here we present the case of a 73-year-old woman who presented with an enlarging umbilical mass, initially thought to be a metastatic lesion, but the imaging and endoscopic exams showed no other primary tumor. So a biopsy was performed, which revealed a localized eccrine porocarcinoma. After a multidisciplinary decision, the tumor was resected with free margins and no additional treatment was needed. The patient remains in follow-up, with no signs of local recurrence nor nodal or distant metastasis.

## Introduction

Eccrine porocarcinoma (EPC) is a rare, slow-growing skin tumor that arises in the eccrine sweat glands and accounts for 0.005%-0.01% of all cutaneous malignancies [1,2]. The tumor was first described in 1963 by Pinkus and Mehregan [3], who named it epidermotropic eccrine carcinoma. However, the term “eccrine porocarcinoma” was only used for the first time in 1969 by Mishima and Morika [4,5] and is currently the most commonly used term. The etiology of this tumor remains unclear. Some researchers hypothesize that it occurs de novo or through the malignant transformation of a long-standing benign eccrine poroma [6,7]. It is acknowledged that there are certain recognized risk factors for the development of these tumors. These include chronic exposure to ultraviolet radiation, immunosuppression and previous exposure to radiotherapy [2]. The clinical course may be aggressive, with a high tendency for recurrence even after wide excision and the potential for distant metastasis [8].

The incidence of EPC increases with age, generally occurring during the sixth to eighth decades of life, with a slight predominance in men [1,2]. Despite its rarity, considerable inconsistency exists in the literature regarding the most common sites of presentation for this malignant tumor. Some authors report a higher incidence in lower extremities [9], while others report on head and neck [10]. In a meta-analysis, Salih et al. [10] reported a higher frequency in head and neck (39.9%), followed by lower extremity (33.9%), upper extremity (8.8%), back (5.1%) and chest wall (4.6%). All studies agree that abdominal presentation is of extreme rarity (2.6%).

EPC is often misdiagnosed due to its varied symptoms. It typically manifests as a nodule with a dome-shaped appearance on dermoscopy, exhibiting thin vascular patterns reminiscent of poroma. It may be asymptomatic or accompanied by pruritus, pain, or bleeding [1]. The mean diameter of the tumor at initial presentation is 2 cm, with a potential range of up to 20 cm [1].

When detected in the early stages, EPC is typically associated with a favorable prognosis. The standard treatment for localized EPC is wide surgical resection. However, the estimated recurrence rate is 20%, while 20% of patients develop lymph node metastasis and 10% have distant metastasis [1,2]. In advanced cases, the prognosis is poor, and in non-resectable cases, the alternative is radiotherapy, chemotherapy or immunotherapy. However, there are few studies regarding these treatment modalities due to the rarity of this tumor [1].

We report a case of an elderly woman with EPC, presenting as an ulcerated nodule in the umbilical region. She was treated with surgical resection of the lesion, with good outcomes.

## Case presentation

A 73-year-old woman attended her general practitioner with a complaint of an enlarging umbilical mass, which had only recently come to her attention. The patient's past medical history included obesity and arterial hypertension. The patient's surgical and family histories were deemed irrelevant.

An ultrasound examination prompted the suspicion of a sister Mary Joseph nodule. Given the normal results of the endoscopy and colonoscopy, a second opinion was sought from the Department of Dermatology at Leiria Hospital Centre.

**Figure 1:**
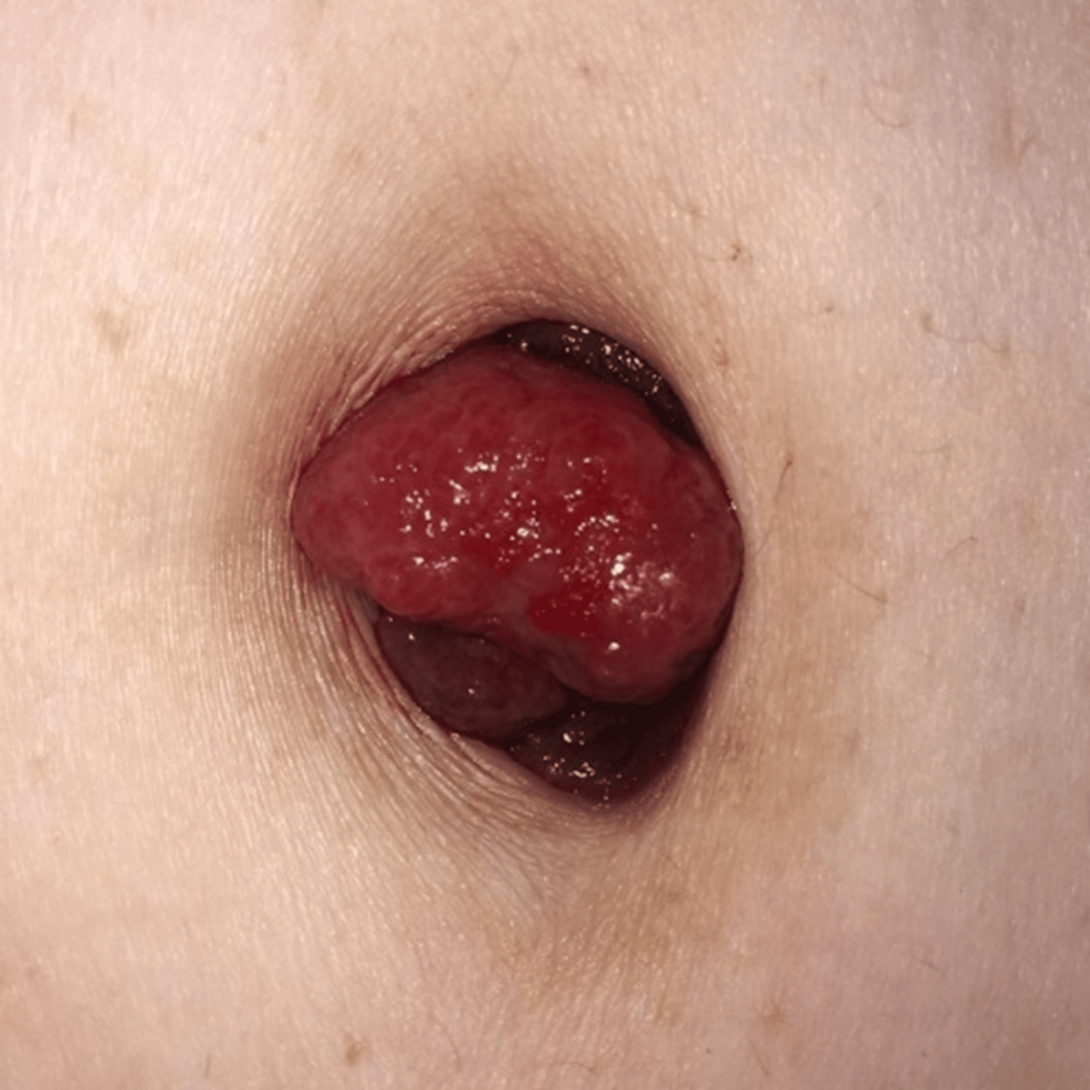
Umbilical erythematous nodule

**Figure 2:**
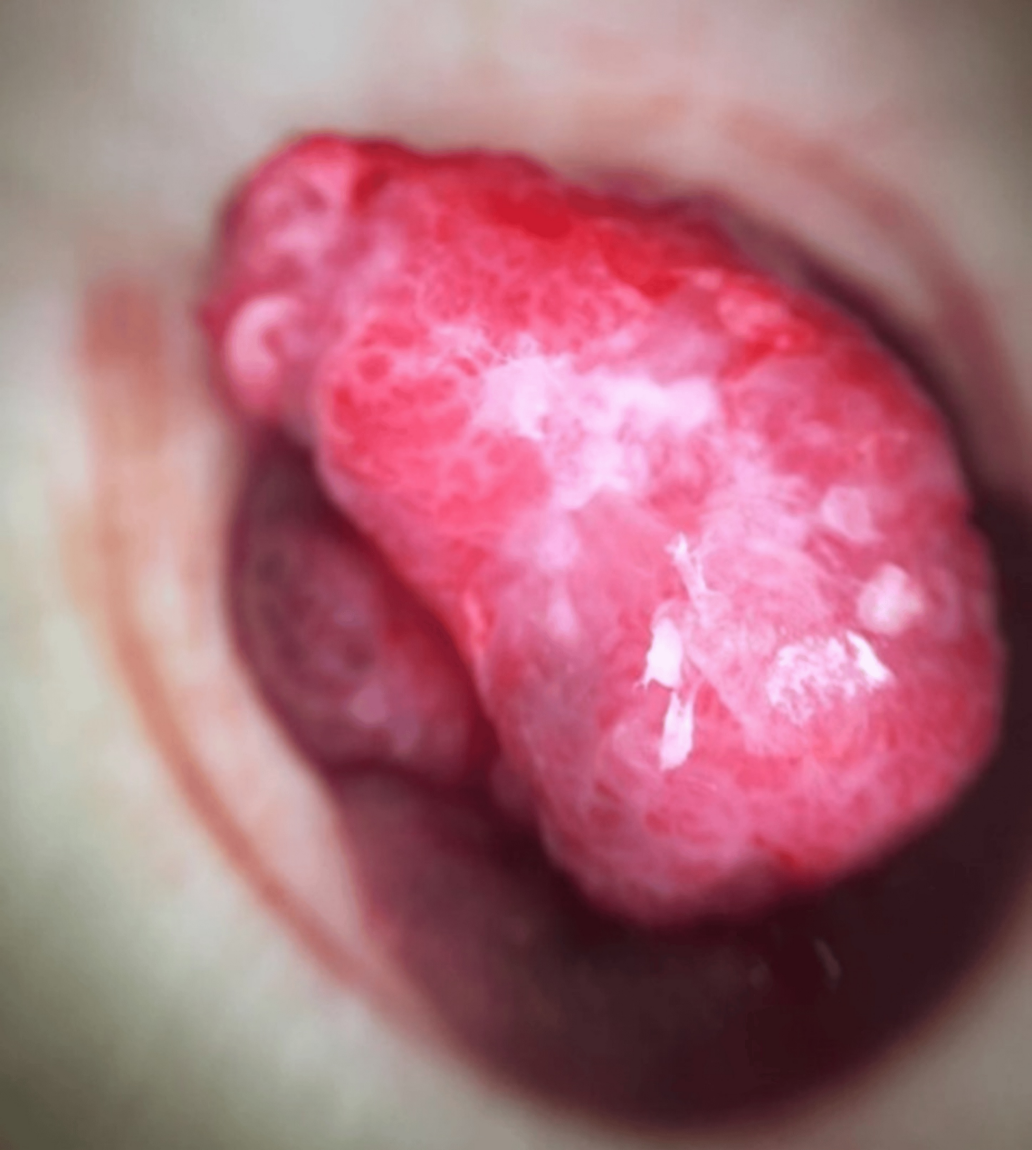
Dermatoscopy of the umbilical nodule

By the time the mass was first examined by the dermatologist, it had grown in size and was erythematous, nodular, firm and non-mobile, with a foul odor and discharge (Figure 1). Dermoscopy of the lesion under polarised light revealed a non-melanocytic lesion with an erythematous background, polymorphic vessels and white streaks (Figure 2).

**Figure 3:**
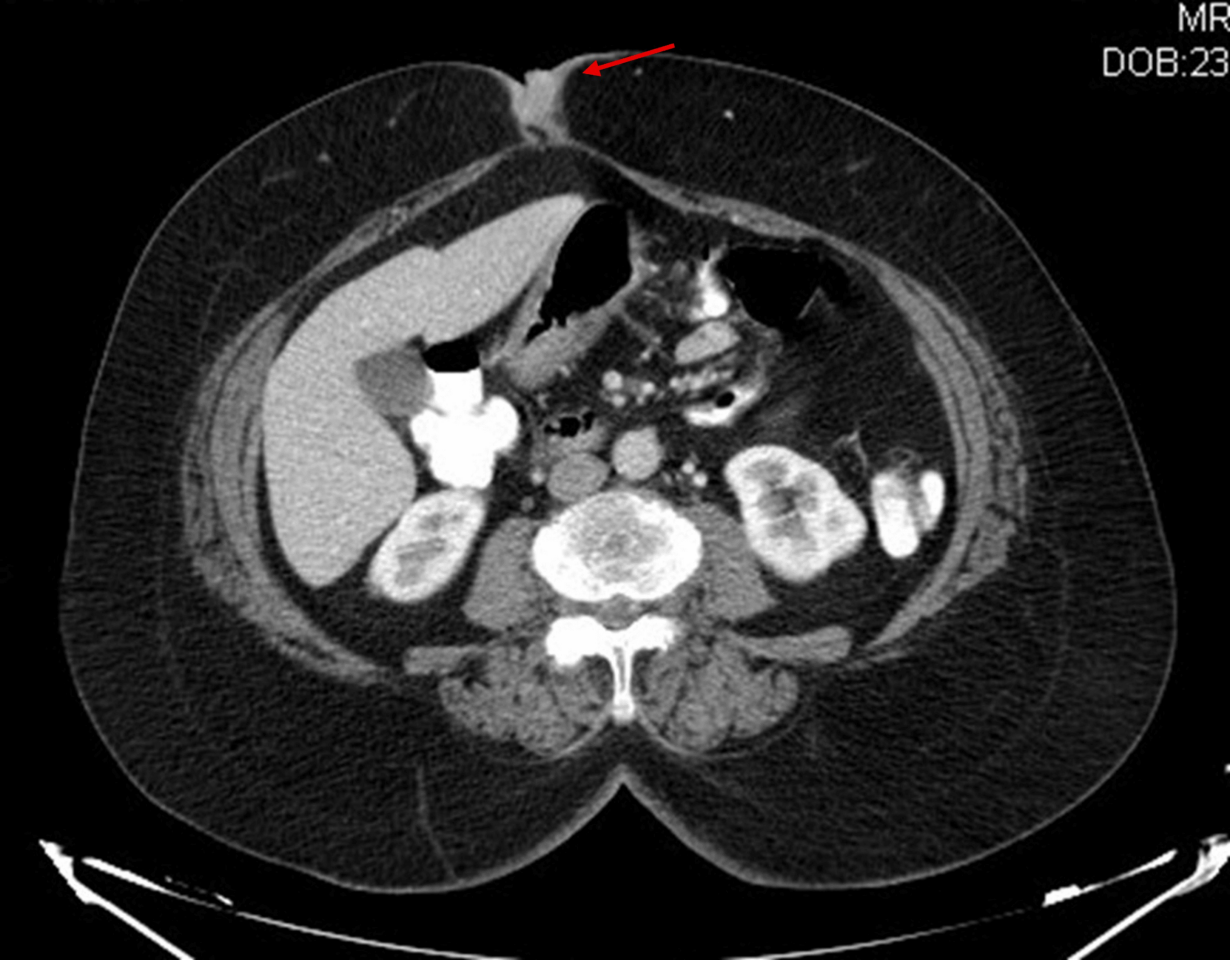
CT scan of the abdomen with contrast (axial view), showing the umbilical mass

A histologic sample was therefore obtained for diagnostic purposes, and the result showed a malignant adnexal skin tumour compatible with an EPC. Computed tomography (CT) scan showed no other abnormalities besides the umbilical lesion (Figure 3). The case was discussed by a multidisciplinary team, which proposed an oncological resection.

**Figure 4:**
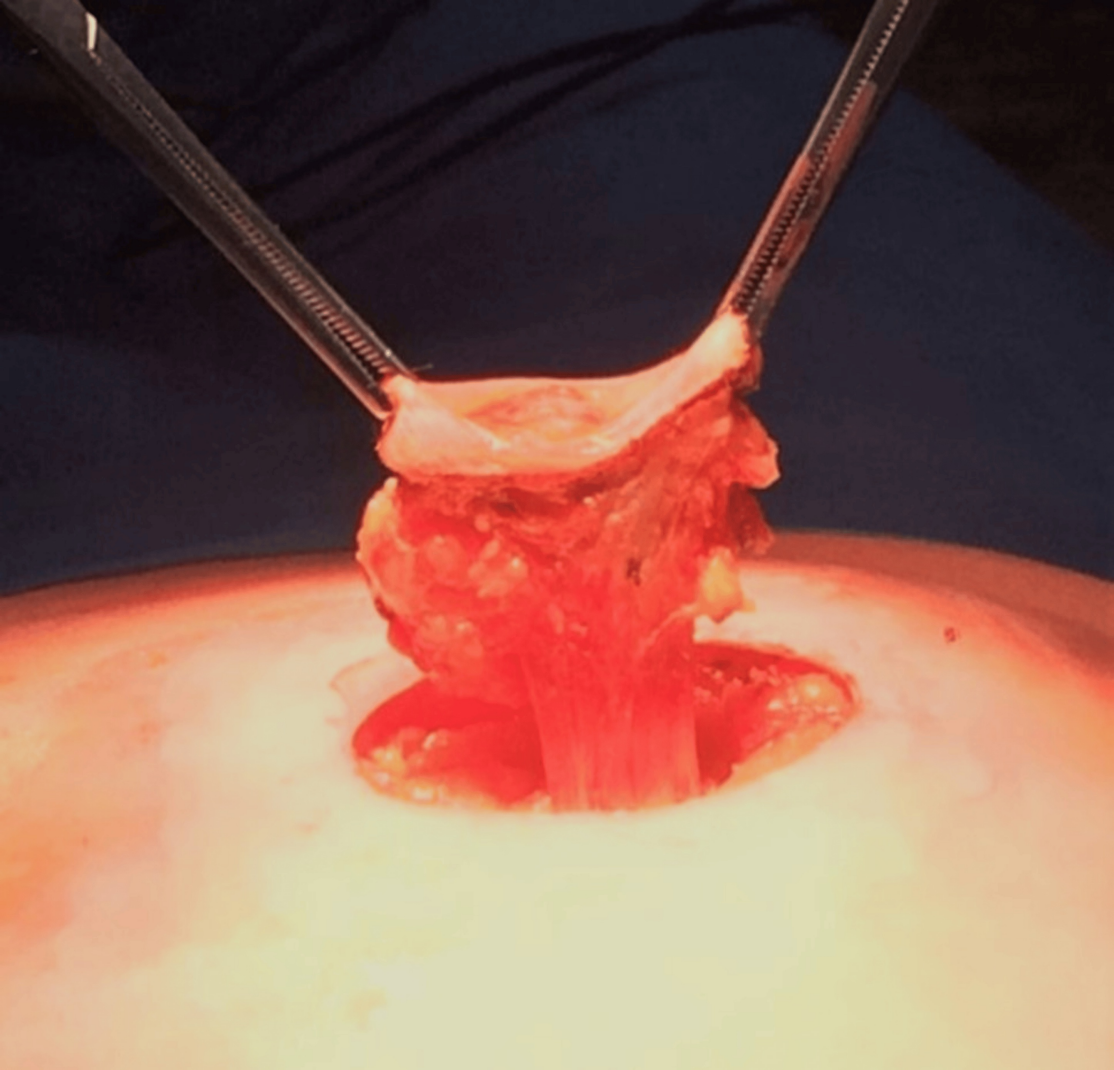
During surgery - total excision of the umbilicus, with free margins

**Figure 5:**
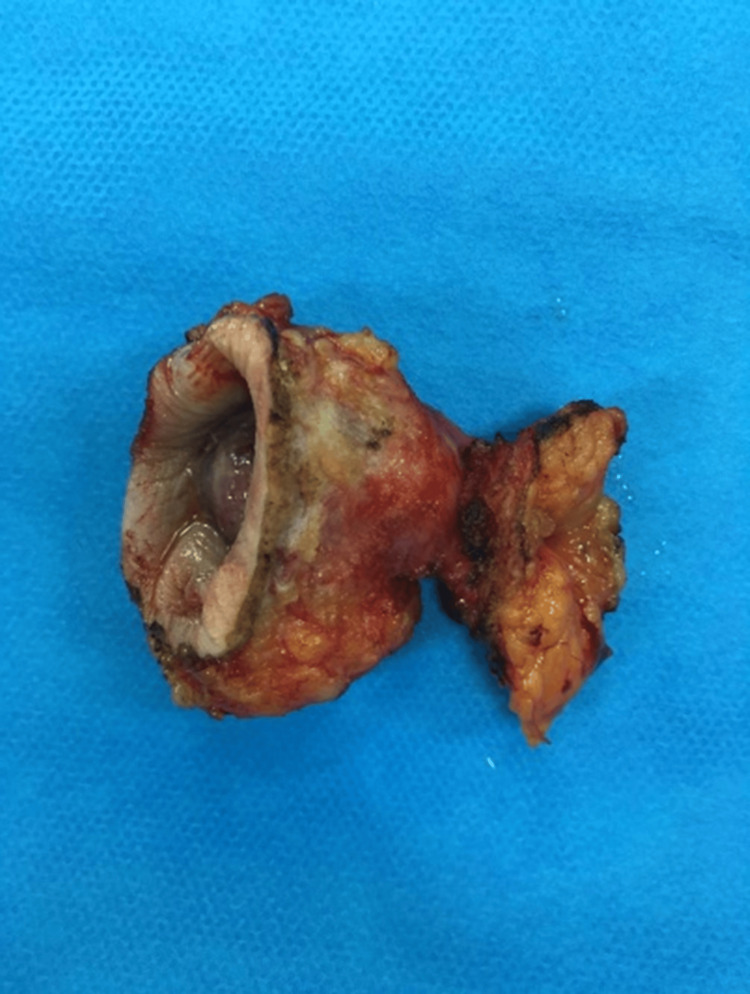
Surgical specimen

Under general anesthesia, radical excision of the tumor with negative margins was performed (Figures 4, 5). The postoperative period (two days) was uneventful.

The pathology examination confirmed an EPC with free resection margins (of 3 cm), low mitotic rate (<14 per high-power field) and no lymphovascular invasion. There was no evidence of recurrence after five months of follow-up.

**Figure 6:**
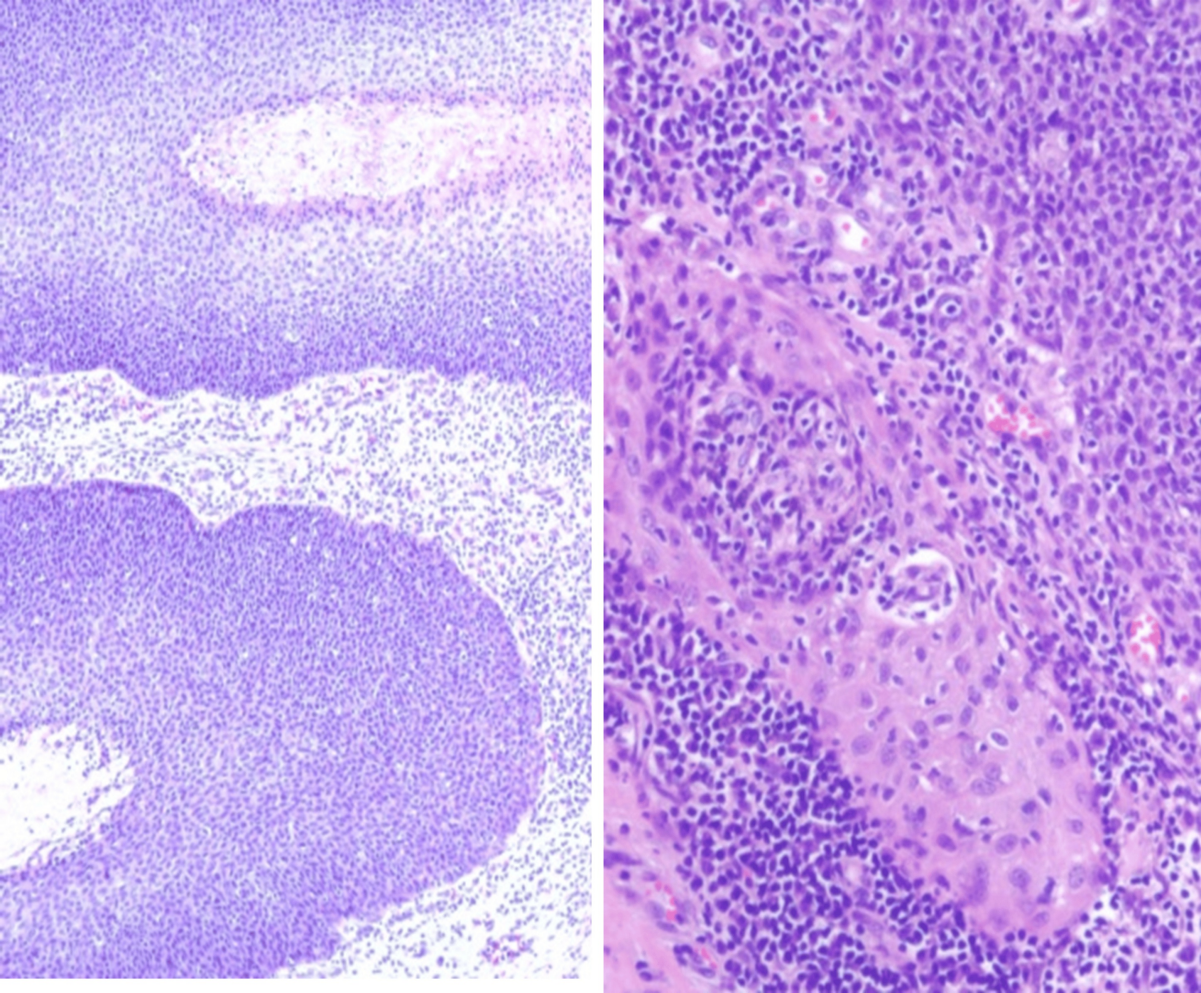
Histopathological image of EPC with atypical cell proliferation and duct formation. Hematoxylin and eosin, original magnification ×10 (right) and x16 (left)

## Discussion

EPC is a rare malignant tumor arising from the eccrine sweat glands, accounting for less than 0.01% of skin tumors. The exact etiology is unknown and it is more common in older people, with a slight male predominance. The most common sites are the head and neck, followed by the lower limbs [11]. The abdomen, as in this case, is a rare site.

It is an aggressive disease with a high rate of recurrence and metastasis even after resection [8]. In advanced stages, the reported mortality rate is 67% [12]. There are reports of patients dying a few months after diagnosis regardless of treatment [13,14]. Due to its rarity, there is controversy surrounding both its presentation and management [15,16].

Clinical presentation can be highly variable. The literature shows that most cases of EPC present with a long-standing mass or nodule [17]. In the study by Kurashige and colleagues [18], all cases of porocarcinoma presented with erosive reddish nodules, whereas Lloyd et al. [16] showed that features such as rapid increase in size, foul odor and fleshy appearance are part of the spectrum of EPC presentation.

Diagnosis can be difficult because EPC may mimic other benign or malignant cutaneous lesions such as pyogenic granuloma, squamous cell carcinoma, basal cell carcinoma, Bowen's disease, Merkel cell carcinoma or extramammary Paget's disease [2]. Dermoscopy may show typical features such as polymorphic or atypical vessels, white globular structures and milky red globules [11]. Tissue biopsy is essential for diagnosis and the characteristic histological findings include mature duct formation and cellular atypia as seen in this case [11,19,20]. There is usually also an increased mitotic rate, tumor necrosis and an infiltrative growth pattern [2]. Although not routinely performed, immunohistochemistry is important for the correct diagnosis, especially when in doubt.

Wide local excision with clear margins is the most commonly used treatment approach for primary EPC [21]. Although regional lymph nodes are the primary site of metastatic disease [22], there is insufficient data to support routine lymph node dissection [10].

Tsunoda et al. conducted a study in 2019, which aimed to access the utility of sentinel lymph node (SLN) biopsy in patients with EPC [23]. They found SLN metastases in 37.5% of cases and, based on this finding, suggested that SLN biopsy followed by lymph node dissection in positive cases may detect lymph node metastases early and thus improve the overall survival of these patients [2,23]. As many as 10% of these patients will develop distant metastases, most commonly to the lung, followed by the liver, brain and skin [1,2,11]. The risk of distant metastases correlates with the anatomical distribution of EPCs - higher in the genital area, followed by the upper limbs, head and neck, and lower limbs [11].

Currently, there are no guidelines on the use of radiotherapy and chemotherapy in the treatment of EPC. Although they are widely used, the results are uncertain. Radiotherapy is used for recurrent and locally advanced disease, positive margins after resection, and high-grade histology with perineural invasion [2]. Chemotherapy is rarely used for primary EPC and is usually reserved for nodal or distant metastases and recurrent disease [11,12]. Immunotherapy is a novel therapy with promising results for advanced or metastatic EPC. Some studies show efficacy of treatment with anti-programmed cell death-1 (PD-1) agents such as pembrolizumab or nivolumab, but further studies are needed to assess their safety and efficacy [2,12].

In this study, EPC was a low-grade, localized tumor treated with wide surgical resection. After histopathological analysis of the specimen, we verified that the margins were negative, with no lymphovascular or perineural invasion, so no additional treatment strategies were needed and the patient entered a follow-up program.

## Conclusions

EPC is a diagnostic challenge due to its rarity and inconsistent location and morphology at presentation. A high index of suspicion is required to diagnose EPC. This case report presents a new site for this rare neoplasm and should be considered in the differential diagnosis of umbilical nodules or masses.

There are no standard protocols or guidelines for its diagnosis and management. Diagnosis is made by histopathological examination and wide surgical excision with histologically clear margins leads to excellent results. There is no clear evidence for regional lymph dissection and adjuvant therapy.
